# Supplementary material for: Effects of SGLT-2 inhibitors on renin-angiotensin-aldosterone system and their correlation with glucose metabolism in type 2 diabetes mellitus patients with hypertension: A prospective study
Source: PLoS One. 2025 Nov 18;20(11):e0336158. doi: 10.1371/journal.pone.0336158 (PMC12626264; doi:10.1371/journal.pone.0336158)
Supplement: S1 Table — (DOCX) [file pone.0336158.s001.docx]

S1 Table. The influence of SGLT-2is treatment on physical and biochemical indicators in DKD patients

| Variables | baseline | 3 months | *p* value |
| --- | --- | --- | --- |
| age（y） | 53.27±14.41 | NA | NA |
| Diabetic duration (M) | 81.0(22.0~213.5) | NA | NA |
| BMI（kg/m^2^） | 26.91(24.94~31.22) | 25.93(24.13~29.49) | <0.001 |
| HbA1c（%） | 9.47±1.82 | 7.86±1.03 | <0.001 |
| FBG（mmol/L） | 10.68±3.12 | 8.04±1.16 | <0.001 |
| TG（mmol/L） | 2.13(1.33~3.87) | 1.51(1.16~2.47) | <0.001 |
| TCHO（mmol/L） | 4.90±1.07 | 4.49±0.71 | 0.003 |
| HDL（mmol/L） | 1.10±0.26 | 1.11±0.19 | 0.670 |
| LDL（mmol/L） | 2.86±0.95 | 2.80±0.75 | 0.675 |
| Urea（mmol/L） | 6.11±2.21 | 6.37±1.47 | 0.285 |
| Crea（μmol/L) | 70.39±28.78 | 73.29±14.43 | 0.414 |
| K（mmol/L） | 4.06±0.27 | 4.09±0.44 | 0.687 |
| Na（mmol/L） | 140.56±2.70 | 140.41±2.44 | 0.692 |
| Ca（mmol/L） | 2.33±0.14 | 2.33±0.12 | 0.969 |
| P（mmol/L） | 1.19±0.19 | 1.18±0.15 | 0.721 |
| SBP（mmHg） | 156.9±21.51 | 147.88±10.93 | <0.001 |
| DBP（mmHg） | 93.19±10.86 | 89.73±7.68 | 0.009 |
| UACR（mg/g） | 87.55(47.34~215.8) | 38.30(15.30~102.91) | <0.001 |
| CP（nmol/L） | 0.65(0.38~1.00) | 0.67(0.44~0.91) | 0.122 |
| HOMA-β（%） | 35.00(22.85~47.75) | 47.60(36.95~69.35) | <0.001 |
| HOMA-IR | 2.03(1.08~3.02) | 1.73(1.14~2.37) | <0.001 |

Data are expressed as “mean ± standard deviation” or “median with interquartile range”. NA, not applicable; SGLT-2is, sodium-glucose cotransporter-2 inhibitors; BMI, body mass index; HbA1c, Hemoglobin A1c; FBG, fast blood glucose; TG, triglycerides; TCHO, total cholesterol; HDL, high-density lipoprotein cholesterol; LDL, low-density lipoprotein cholesterol; Urea, urea nitrogen; Crea, creatinine; K, potassium; Na, sodium; Ca, calcium; P, phosphorus; SBP, systolic blood pressure; DBP, diastolic blood pressure; UACR, urinary albumin-to-creatinine ratio; CP, C-peptide; HOMA-β, Homeostasis Model Assessment of β-cell function ; HOMA-IR, Homeostasis Model Assessment of insulin resistance.
