# Supplementary material for: Effects of SGLT-2 inhibitors on renin-angiotensin-aldosterone system and their correlation with glucose metabolism in type 2 diabetes mellitus patients with hypertension: A prospective study
Source: PLoS One. 2025 Nov 18;20(11):e0336158. doi: 10.1371/journal.pone.0336158 (PMC12626264; doi:10.1371/journal.pone.0336158)
Supplement: S2 Table — (DOCX) [file pone.0336158.s002.docx]

S2 Table. The influence of SGLT-2is treatment on physical and biochemical indicators in non-DKD patients

| Variables | baseline | 3 months | *p* value |
| --- | --- | --- | --- |
| age（y） | 55.76±12.17 | NA | NA |
| Diabetic duration (M) | 72.0(12.0~135.0) | NA | NA |
| BMI（kg/m^2^） | 26.72(24.41~29.26) | 25.80(23.61~28.29) | <0.001 |
| HbA1c（%） | 9.08±2.29 | 7.77±1.16 | <0.001 |
| FBG（mmol/L） | 9.40±2.69 | 7.70±1.18 | <0.001 |
| TG（mmol/L） | 1.58(1.15~2.62) | 1.24(0.98~1.90) | <0.001 |
| TCHO（mmol/L） | 4.86±1.34 | 4.38±0.64 | 0.002 |
| HDL（mmol/L） | 1.07±0.29 | 1.09±0.18 | 0.496 |
| LDL（mmol/L） | 2.93±1.09 | 2.73±0.64 | 0.078 |
| Urea（mmol/L） | 5.55±1.31 | 5.69±1.20 | 0.443 |
| Crea（μmol/L) | 60.26±15.26 | 64.39±11.59 | 0.032 |
| K（mmol/L） | 4.06±0.46 | 4.08±0.30 | 0.726 |
| Na（mmol/L） | 140.84±2.62 | 140.79±2.17 | 0.909 |
| Ca（mmol/L） | 2.33±0.13 | 2.33±0.11 | 0.956 |
| P（mmol/L） | 1.19±0.15 | 1.18±0.14 | 0.694 |
| SBP（mmHg） | 153.07±18.02 | 146.85±12.74 | 0.002 |
| DBP（mmHg） | 92.86±11.05 | 89.20±8.12 | 0.010 |
| UACR（mg/g） | 10.19(6.10~14.81) | 5.30(3.20~8.98) | <0.001 |
| CP（nmol/L） | 0.69(0.43~0.91) | 0.64(0.48~0.85) | 0.212 |
| HOMA-β（%） | 43.00(27.85~58.10) | 56.30(41.15~68.25) | <0.001 |
| HOMA-IR | 1.86(1.08~2.49) | 1.62(1.16~2.14) | 0.004 |

Data are expressed as “mean ± standard deviation” or “median with interquartile range”. NA, not applicable; SGLT-2is, sodium-glucose cotransporter-2 inhibitors; BMI, body mass index; HbA1c, Hemoglobin A1c; FBG, fast blood glucose; TG, triglycerides; TCHO, total cholesterol; HDL, high-density lipoprotein cholesterol; LDL, low-density lipoprotein cholesterol; Urea, urea nitrogen; Crea, creatinine; K, potassium; Na, sodium; Ca, calcium; P, phosphorus; SBP, systolic blood pressure; DBP, diastolic blood pressure; UACR, urinary albumin-to-creatinine ratio; CP, C-peptide; HOMA-β, Homeostasis Model Assessment of β-cell function ; HOMA-IR, Homeostasis Model Assessment of insulin resistance.
