# Supplementary material for: Effects of SGLT-2 inhibitors on renin-angiotensin-aldosterone system and their correlation with glucose metabolism in type 2 diabetes mellitus patients with hypertension: A prospective study
Source: PLoS One. 2025 Nov 18;20(11):e0336158. doi: 10.1371/journal.pone.0336158 (PMC12626264; doi:10.1371/journal.pone.0336158)
Supplement: S3 Table — (DOCX) [file pone.0336158.s003.docx]

S3 Table. Comparison analysis of RAAS and other biochemical indicators between DKD and non-DKD patients after 3-month treatment.

| Variables | DKD | non-DKD | *p* value |
| --- | --- | --- | --- |
| Diabetic duration (M) | 84.0(25.0~216.5) | 75.0(15.0~138.0) | 0.101 |
| BMI（kg/m^2^） | 25.93(24.13~29.49) | 25.80(23.61~28.29) | 0.228 |
| HbA1c（%） | 7.86±1.03 | 7.77±1.16 | 0.642 |
| FBG（mmol/L） | 8.04±1.16 | 7.70±1.18 | 0.081 |
| TG（mmol/L） | 1.51(1.16~2.47) | 1.24(0.98~1.90) | 0.012 |
| TCHO（mmol/L） | 4.49±0.71 | 4.38±0.64 | 0.298 |
| HDL（mmol/L） | 1.11±0.19 | 1.09±0.18 | 0.452 |
| LDL（mmol/L） | 2.80±0.75 | 2.73±0.64 | 0.524 |
| Urea（mmol/L） | 6.37±1.47 | 5.69±1.20 | 0.003 |
| Crea（µmol/L) | 73.29±14.43 | 64.39±11.59 | <0.001 |
| K（mmol/L） | 4.09±0.44 | 4.08±0.30 | 0.940 |
| Na（mmol/L） | 140.41±2.44 | 140.79±2.17 | 0.322 |
| Ca（mmol/L） | 2.33±0.12 | 2.33±0.11 | 0.779 |
| P（mmol/L） | 1.18±0.15 | 1.18±0.14 | 0.995 |
| SBP（mmHg） | 147.88±10.93 | 146.85±12.74 | 0.602 |
| DBP（mmHg） | 89.73±7.68 | 89.20±8.12 | 0.689 |
| UACR（mg/g） | 38.30(15.30~102.91) | 5.30(3.20~8.98) | <0.001 |
| AII（ng/L） | 107(97~121) | 115(103~121) | 0.176 |
| ALD（ng/L） | 141(105~173) | 138(110~158) | 0.866 |
| REN（ng/L） | 13.90(6.85~22.85) | 14.35(9.68~21.83) | 0.123 |
| ARR | 1.15(0.75~1.77) | 0.88(0.63~1.35) | 0.030 |
| CP（nmol/L） | 0.67(0.44~0.91) | 0.64(0.48~0.85) | 0.883 |
| HOMA-β（%） | 47.60(36.95~69.35) | 56.30(41.15~68.25) | 0.257 |
| HOMA-IR | 1.73(1.14~2.37) | 1.62(1.16~2.14) | 0.663 |
|  |  |  |  |

Data are expressed as “mean ± standard deviation” or “median with interquartile range”. DKD, diabetic kidney disease; HbA1c, Hemoglobin A1c; FBG, fast blood glucose; TCHO, total cholesterol; HDL, high-density lipoprotein cholesterol; LDL, low-density lipoprotein cholesterol; Urea, urea nitrogen; Crea, creatinine; K, potassium; Na, sodium; Ca, calcium; P, phosphorus; SBP, systolic blood pressure; DBP, diastolic blood pressure; AII, angiotensin II; ALD, aldosterone; REN, renin; ARR, aldosterone-to-renin ratio; CP, C-peptide; HOMA-β, Homeostasis Model Assessment of β-cell function; HOMA-IR, Homeostasis Model Assessment of insulin resistance; UACR, urinary albumin-to-creatinine ratio; TG, triglycerides; BMI, body mass index.
