# Supplementary material for: Effects of SGLT-2 inhibitors on renin-angiotensin-aldosterone system and their correlation with glucose metabolism in type 2 diabetes mellitus patients with hypertension: A prospective study
Source: PLoS One. 2025 Nov 18;20(11):e0336158. doi: 10.1371/journal.pone.0336158 (PMC12626264; doi:10.1371/journal.pone.0336158)
Supplement: Attachment — Submitted filename: Response to reviewers-PONE-D-25-15425R1(20250801).docx [file pone.0336158.s005.docx]

**2025.07.31**

Dear Daniele Romanello:

**Re:** **PONE-D-25-15425**

**Title:** Effects of SGLT-2 inhibitors on renin-angiotensin-aldosterone system and their correlation with glucose metabolism in type 2 diabetes mellitus patients with hypertension: A prospective study

Thank you for your kind letter of “**PLOS ONE PONE-D-25-15425 Revision Request**” on **June 02, 2025**. We thank the editorial requests for the corrections, and we deeply apologized for the considerable inconvenience caused to you due to our errors exist. We have now revised the manuscript according to the concerns raised in the reviews. A detailed copy of the authors' “point-by-point response to the reviewer's comments” is attached, and specific changes addressing reviewers' comments are marked.

Again, we would like to thank you for taking the time to review our manuscript. We would be most grateful for your kind consideration of this revised manuscript for potential publication in ***PLOS ONE***.

We look forward to hearing from you at your earliest convenience.

Sincerely yours,

Ningning Wang

Corresponding author:

Baohong Yue, Professor, PhD

Address: Department of Laboratory Medicine, The First Affiliated Hospital of Zhengzhou University, Zhengzhou, P. R. China

**Responses to the Reviewer’s comments:**

**Re: PONE-D-25-15425**

**Title:** Effects of SGLT-2 inhibitors on renin-angiotensin-aldosterone system and their correlation with glucose metabolism in type 2 diabetes mellitus patients with hypertension: A prospective study

We would like to acknowledge the reviewer’s insightful comments and valuable suggestions. In addition, we thank the reviewer for helping us improve the present manuscript. We have addressed the concerns raised by the reviewer as detailed below.

**Reviewer #1**

**C1:** SGLT2i are cornerstone of the management of T2DM. They are very important tools in delaying the progression of chronic kidney disease and reducing the risk of hospitalization for heart failure. therefore, it is important to understand their mechanism of action and the effect on other pathophysiological mechanisms of complications. the study is an important study.

**A1:** We are appreciated to note the favorable comments of reviewer in the comments.

**C2:** the number of the studied patients is too small for such study to make the results credible. More important it is known that SGLT2i results in early short-term rise in serum creatinine and drop of creatinine clearance followed by slowing of the deterioration of kidney function; a study for one month may give results different from long term studies as shown in previous studies.

**A2:** Great thanks for your comment. We have extended the follow-up period by two months, and “patients were prescribed 10 mg dapagliflozin daily for 3 months. The RAAS and other relevant biomarkers were measured and compared during outpatient visits at baseline, 1 month, and 3 months”. All data, tables, and graphs have been thoroughly reorganized and reanalyzed. The results, abstract, introduction and discussion sections have been revised accordingly.

**C3:** It is better to use DKD instead of DN which may be interpreted as diabetic neuropathy. The reference for "diagnostic criteria for diabetic complications" should be mentioned in the methods.

**A3:** Great thanks for your comment. We have used DKD instead of DN, and the entire document has been thoroughly reviewed and revised. The reference [23] for "diagnostic criteria for diabetic complications" has been added to Section ***2.1*** of the Methods.

**C4:** promoting the excretion of glycosuria is better to be replaced by increasing glycosuria. preventing β-cell failure is a wishful thinking rather than evidence-based effect. So, it is better to use do not increase beta cell apoptosis (which is an inevitable part of the diabetic syndrome).

**A4:** Great thanks for your comment. We have revised “promoting the excretion of glycosuria” to “increasing glycosuria” and revised “preventing β-cell failure” to “slowing β-cell failure”.

**C5:** how did you diagnose albuminuria, I thing single random test is not enough and 2 positive tests out of three are needed.

**A5:** Great thanks for your comment. Yes, urinary albumin was evaluated by collecting three midstream urine samples over three consecutive days. The UACR exceeding 30 mg/g in two out of three tests indicated the presence of microalbuminuria.

**Reviewer #2**

**C1:** The study addresses an important and underexplored interaction between SGLT-2 inhibitors, RAAS, and glucose metabolism in T2DM with implications for primary aldosteronism (PA) is commendable.

**A1:** We are appreciated to note the favorable comments of reviewer in the comments.

**C2:** Detailed evaluation of multiple metabolic and RAAS components (e.g., ALD, REN, ARR, HOMA indices) provides a robust biochemical profile.

**A2:** We are appreciated to note the favorable comments of reviewer in the comments.

**C3:** A one-month treatment duration is insufficient to draw long-term conclusions, especially regarding ARR variability and its implications for PA screening.
**A3:** Great thanks for your comment. We have extended the follow-up period by two months, and “patients were prescribed 10 mg dapagliflozin daily for 3 months. The RAAS and other relevant biomarkers were measured and compared during outpatient visits at baseline, 1 month, and 3 months”. All data, tables, and graphs have been thoroughly reorganized and reanalyzed. The results, abstract, introduction and discussion sections have been revised accordingly.

**C4:** Further explanation is needed on how short-term SGLT-2i-induced ARR changes might clinically mislead PA diagnosis.
**A4:** Great thanks for your comment. We have elaborated further explanation in the **second and third** paragraphs of the ***Discussion***, and the conclusions have been updated accordingly. For example, “However, short-term therapy (≤3 months) may elevate REN levels and reduce ARR. Consequently, clinicians should exercise caution when interpreting ARR in T2DM patients on short-term SGLT-2is therapy due to the risk of false-negative PA screening. Further studies to determine the optimal duration for discontinuing SGLT-2is before ARR test are warranted and should validate these findings in PA patients”.

**C5:** Heatmaps and regression tables should include clearer legends and color scales for easier interpretation.

**A5:** Great thanks for your comment. Heatmaps and regression tables have been revised to facilitate easier interpretation.

**C6:** This manuscript would benefit from proof reading and simplifying complex sentences improve the manuscript’s readability.

**A6:** Great thanks for your comment. We have carefully revised the text, and an English language editor has edited this study. The revised parts were highlighting in the manuscript.

**C7:** While DN and non-DN groups are compared, additional stratification based on baseline ARR or insulin resistance could provide deeper insights.
**A7:** Great thanks for your comment. This article conducted both inter-group horizontal comparisons and longitudinal analyses over time. Additionally, a correlation analysis between RAAS and glucose metabolism indicators was performed. Due to length constraints, further stratification based on baseline ARR and IR was not included. This is a valuable suggestion; however, in this study, an additional comparison of temporal differences was incorporated when comparing DKD and non-DKD groups. The results are as follows: In ***3.2*** section：“Additionally, the differences from the baseline in FBG and UACR values were significantly higher in DKD group than those in non-DKD group (Table 2).” “After 3 months of dapagliflozin administration, the levels of TG (1.51 vs. 1.24 mmol/L), Crea (73.29 vs. 64.39 µmol/L), and UACR (38.30 vs. 5.30) in DKD group remained significantly higher than those in non-DKD group (p<0.05, S3 Table).” In ***3.3*** section：“However, the increase in REN levels from baseline to 1 month was significantly greater in the DKD group compared to the non-DKD group (4.15±7.35 vs. 2.75±8.03 ng/L; DKD vs. non-DKD; p<0.05).”

**C8:** There are no mention of statistical power or sample size estimation raises concerns about the adequacy of the sample to detect differences.
**A8:** Great thanks for your comment. In the statistical analysis, methods for estimating sample size have been included. The statement is as follows: “We calculated that sample size of 64 was necessary to provide up to 80% power to detect a difference in mean REN level between two groups, assuming a mean difference of 0.5 ng/L and standard deviation of 1.0 ng/L according to previous study with significance of 0.05 and power of 80%. Hence, we set the sample size to 147.”

**C9:** The availability of raw data in a public repository is asserted, but the actual link or repository is not provided.

**A9:** Great thanks for your comment. The raw data has been uploaded to the public repository available at https://github.com/ningningwang688/SGLT-2is-on-T2DM.

**Reviewer #3**

**C1:** The authors have done an excellent job of selecting a highly relevant and impactful research topic and has presented it in a clear and engaging manner. This demonstrates both a good understanding of the field and a commitment to addressing an important area of inquiry. However, I would like to raise certain queries and thoughts as regards the manuscript.

**A1:** We are appreciated to note the favorable comments of reviewer in the comments.

**C2:** Title: The current title is informative and captures the study’s scope, but its length and vagueness in certain terms (e.g., “exploring the interaction”) reduce its impact. The title does not indicate whether the study is observational, experimental, or prospective, which could help readers quickly understand the study’s methodology and rigor. Refining the title to be more concise, precise, and aligned with the study’s findings—particularly by clarifying the speculative nature of PA implications—will enhance its appeal to readers and better reflect the study’s contribution.

**A2:** Great thanks for your comment. We have revised the title as “Effects of SGLT-2 inhibitors on renin-angiotensin-aldosterone system and their correlation with glucose metabolism in type 2 diabetes mellitus patients with hypertension: A prospective study”

**C3:** Introduction: The introduction is too lengthy and doesn’t bring out effectively the gap and rationale for this research. It would be suggested that authors could work and incorporate these aspects into introduction to make it more crisp.

**A3:** Great thanks for your comment. We have revised the introduction, simplified the sentences, and summarized the main points.

**C4:** Aim and objective: The aim is vague, the objective is overambitious with the claim about PA diagnosis strategies, and there is lack of specificity regarding the SGLT-2i agent. Recommended to revise the aim and objective to be more clearly worded, concise and specific.

**A4:** Great thanks for your comment. We have revised the objective as “Consequently, this prospective self-matched study was undertaken to investigate the effects of SGLT-2is on RAAS and their associations with glucose metabolism, aiming to improve the clinical interpretation of ARR test in patients with T2DM and hypertension.”

Methodology:
**C5:** Study design - The selection of the study design is congruent with the aim and objective of this research. But the methodology does not clarify the sampling method (e.g., consecutive, random, or convenience sampling), which could introduce selection bias. It is unclear how patients were identified or approached for recruitment. Authors to specify the sampling strategy to ensure reproducibility and address potential biases.

**A5:** Great thanks for your comment. In ***2.1*** section of the ***Method***, the sampling strategy have been included. The statement is as follows: “Patients diagnosed with T2DM and hypertension were identified and recruited through convenience consecutive sampling at routine diabetes outpatient visits to the First People's Hospital of Pingdingshan between April 2024 and November 2024.”

**C6:** The criteria for diagnosing diabetic nephropathy are referenced as "established diagnostic criteria" but not explicitly described or cited, which reduces transparency. Additionally, the near-identical median diabetic duration (72 months) in both groups seems unusual and may warrant explanation or verification.

**A6:** Great thanks for your comment. The reference [23] for "diagnostic criteria for diabetic complications" has been added to Section ***2.1*** of the ***Methods***. The median duration of diabetes in DKD group was 81 months. We apologize for our errors exist, and have corrected the inaccurate time.

**C7:** The rationale for the one-month treatment duration is not justified, and it is unclear whether this duration is sufficient to observe meaningful changes in RAAS or other indicators. Additionally, there is no mention of monitoring adherence to the treatment or managing potential side effects.

**A7:** Great thanks for your comment. We have extended the follow-up period by two months, and “patients were prescribed 10 mg dapagliflozin daily for 3 months. The RAAS and other relevant biomarkers were measured and compared during outpatient visits at baseline, 1 month, and 3 months”. All data, tables, and graphs have been thoroughly reorganized and reanalyzed. The results, abstract, introduction and discussion sections have been revised accordingly.

We have added the safety assessment. The statements are as follows: In ***2.3*** section of the ***Methods***: “To monitor treatment adherence and manage potential side effects, a safety assessment was conducted based on adverse event reports during the study.”

In ***3.6*** section of the ***Results***: “During the study period, a total of 23 patients (15.6%) experienced 31 adverse events, including 11 cases of thirst, 4 cases of hypoglycemia, 5 cases of genital itching, 6 cases of constipation, 3 cases of skin itching, and 2 cases of pollakiuria. Importantly, no adverse events were classified as severe. A total of 147 patients successfully completed the trial.”

**C8:** The washout period for medications affecting ARR (2–4 weeks) is broad and not standardized, which could introduce variability. The use of terazosin for poorly controlled blood pressure is mentioned, but the criteria for "poorly controlled" are not defined, and its potential impact on study outcomes is not addressed. Additionally, the exclusion of patients with contraindications to dapagliflozin is vague, as specific contraindications are not listed.

**A8:** Great thanks for your comment. We have revised the vague statements. In ***2.2*** section: “Patients had not taken any agents that could markedly affect the ARR, such as ACEIs, ARBs, diuretics, or calcium channel blockers within the last 2 months, or aldosterone receptor antagonists, β-blockers, or direct renin inhibitors within the last 4 months [8]; For patients with poorly controlled blood pressure (≥160/100 mmHg), medications with less effect on RAAS, such as α-blockers (e.g., trazodone), were administered.”

We have included the specific contraindications. In ***2.2*** section: “Patients with contraindications to dapagliflozin therapy (experiencing side effects such as drug hypersensitivity, complicated urinary tract infections or genital infections, and acute kidney injury);”

**C9:** The timing of post-treatment assessments is not clearly stated (e.g., immediately after the one-month treatment or at a specific follow-up point). Additionally, there is no mention of quality control measures for laboratory assays (e.g., calibration, inter-assay variability) or blinding of laboratory personnel to group allocation.

**A9:** Great thanks for your comment. We have added the statements. In ***2.1*** section: “After obtaining written informed consent, eligible patients were prescribed dapagliflozin at a daily dosage of 10 mg for a period of three months. The RAAS and other relevant indicators were measured during outpatient visits at baseline, 1 month, and 3 months.”

In ***2.3*** section: “All tests were processed and analyzed concurrently with the daily specimens according to ISO (International Organisation for Standardisation) 15189: 2012 standards. The laboratory personnel were blinded to the group assignments and patient information.”

**C10:** The methodology does not specify how sample size was determined, which is critical for assessing the study’s power to detect differences.

**A10:** Great thanks for your comment. In the statistical analysis, methods for estimating sample size have been included. The statement is as follows: “We calculated that sample size of 64 was necessary to provide up to 80% power to detect a difference in mean REN level between two groups, assuming a mean difference of 0.5 ng/L and standard deviation of 1.0 ng/L according to previous study with significance of 0.05 and power of 80%. Hence, we set the sample size to 147.”

**C11:** The term “two covariate models” in the regression analysis is unclear, as the covariates included are not specified. Additionally, there is no mention of adjusting for multiple comparisons, which could increase the risk of type I errors given the number of indicators tested.

**A11:** Great thanks for your comment. This study primarily involved comparisons between two independent samples and paired data. Multiple linear regression analysis was performed using the "Enter" method in two covariate models, with adjustments made for multiple variables, so multiple comparisons were unnecessary. In the statistical analysis, sequential multivariable adjustment modeling has been included. Model 1: age, total cholesterol, triglycerides, high-density lipoprotein cholesterol, low-density lipoprotein cholesterol, urea nitrogen, creatinine, potassium, sodium, calcium, phosphorus, systolic blood pressure, diastolic blood pressure, body mass index and diabetic duration. Model 2: Model 1+the RAAS components (Log-REN, ALD or AII).

**C12:** There is no mention of a data monitoring committee or procedures for handling adverse events during the study, which are important for patient safety in clinical trials.

**A12:** Great thanks for your comment. We have added the safety assessment. The statements are as follows: In ***2.3*** section of the ***Methods***: “To monitor treatment adherence and manage potential side effects, a safety assessment was conducted based on adverse event reports during the study.”

In ***3.6*** section of the ***Results***: “During the study period, a total of 23 patients (15.6%) experienced 31 adverse events, including 11 cases of thirst, 4 cases of hypoglycemia, 5 cases of genital itching, 6 cases of constipation, 3 cases of skin itching, and 2 cases of pollakiuria. Importantly, no adverse events were classified as severe. A total of 147 patients successfully completed the trial.”
Results:

**C13:** The text does not provide effect sizes or actual values for the changes in REN and ARR, making it difficult to assess the clinical significance of these findings. Additionally, the lack of comparison between DN and non-DN groups post-treatment (e.g., whether the magnitude of REN increase differed) limits the interpretation of group-specific effects.

**A13:** Great thanks for your comment. We have included the effect sizes and actual values for REN and ARR, and compared the magnitude of the increase in REN. The statements are as follows:

In ***3.3*** section of the results: “REN levels increased significantly at the 1-month visit in both the DKD and non-DKD groups (p<0.001 and p=0.006, respectively) (Fig 3C). However, the increase in REN levels from baseline to 1 month was significantly greater in the DKD group compared to the non-DKD group (4.15±7.35 vs. 2.75±8.03 ng/L; DKD vs. non-DKD; p<0.05).”

“Unlike renin, ARR values decreased significantly from baseline at both the 1- and 3-month visits in both patient groups (p<0.01, Table 3 and Fig 3D). Moreover, the differences from baseline to the 1- or 3-month visits were not significant between the two groups (1 month, -0.70±1.16 vs. -0.59±1.19 ng/L; DKD vs. non-DKD; 3 months, -0.45±1.15 vs. -0.42±1.07 ng/L; DKD vs. non-DKD; p>0.05).”

**C14:** The reporting of statistical significance (e.g., p<0.05) is consistent, but the text does not discuss the clinical relevance of the observed changes (e.g., whether a 1.59% reduction in HbA1c in DN patients is meaningful). The glycosylated hemoglobin was evaluated 1 month after the treatment initiation. While some minor changes in HbA1c may be seen within 4 weeks (lifespan of RBC is 120 days), it typically takes 8-12 weeks to show some meaningful changes. Hence it is difficult to understand the rationale of testing this parameter within 1 month.

**A14:** Great thanks for your comment. This article has extended the follow-up period. The comparison now focuses on the change in HbA1c levels three months later. The discussion and results sections have also been appropriately revised.

**C15:** The mention of significant reductions in Crea (DN group) and TCHO/LDL (non-DN group) is brief and lacks context about their implications. Additionally, the persistent elevation of UACR and TG in the DN group post-treatment is noted but not explored further.

**A15:** Great thanks for your comment. This is explained in the ***fourth*** paragraph of the discussion, as follows: “Patients with DKD exhibited significantly elevated levels of FBG, Crea, UACR and TG, alongside reduced HOMA-β values compared with non-DKD patient at the start of our study. However, no significant differences in RAAS components were observed between the two groups, indicating that blood glucose levels, renal function, and diabetes status did not influence RAAS prior to treatment, enabling comparable results afterward.”

**C16:** The tables include variables like age and diabetic duration, which are not expected to change post-treatment, yet diabetic duration shows a statistically significant increase (p<0.001) in both groups, which seems implausible within a one-month study period and suggests a potential error in data reporting or analysis. But if it’s not an error and authors have tried to incorporate the 1 month of study period, I think it will not be sound practice to do so.

**A16:** Great thanks for your comment. After one treatment, the age and disease duration of all patients increased by one month. Although this change is statistically significant, it lacks practical clinical relevance. Therefore, we deleted these statistical results and labeled them as “not applicable”.

**C17:** Additionally, the tables are dense, and some variables (e.g., K, Na, Ca, P) show no significant changes, which could be streamlined to focus on key findings.

**A17:** Great thanks for your comment. K, Na, Ca and P did not show significant changes. However, since they were necessary for establishing covariate models in multiple linear regression analysis, these data were retained.

**C18:** The text lists multiple correlations but does not provide correlation coefficients (e.g., r values) or their magnitudes, making it difficult to assess the strength of relationships. The loss of certain correlations post-treatment (e.g., ALD and UACR in DN) is noted but not discussed in terms of biological or clinical implications. It would be advisable to report specific correlation coefficients (r values) for significant findings to quantify the strength of associations. Discuss the biological or clinical relevance of key correlations (e.g., why ALD and REN correlate with CP and HOMA-IR) and the loss of correlations post-treatment.

**A18:** Great thanks for your comment. We have revised the statements and added the “r values” in ***3.4*** section of Results, for example: “ALD and REN exhibited positive correlations with CP (r=0.400 and 0.414) and HOMA-IR ((r=0.467 and 0.413), while demonstrating negative correlations with UACR (r=-0.380 and -0.545). In contrast to REN, ARR displayed negative correlations with CP(r=-0.332), and HOMA-IR (r= -0.363), but positive correlation with UACR (r=0.545).” The discussion has also been appropriately revised in the ***fifth*** paragraph of the discussion.

**C19:** The regression results are complex and difficult to interpret due to vague phrasing (e.g., “correlations were not independent of the RAAS and dapagliflozin treatment”). The distinction between Model 1 and Model 2 is not clearly explained in the text, and the rationale for including specific covariates is not provided. Additionally, some p-values in Table 3 are reported as “<0.001” for non-significant results (e.g., Log-ARR in DN pre-treatment), which is confusing and likely a typographical error. It would be advisable to clarify the purpose and differences between Model 1 and Model 2 in the text, specifying which covariates were included and why. Use precise language to describe whether correlations were independent of RAAS components, dapagliflozin, or both.

**A19:** Great thanks for your comment. We have clarified the purpose and differences between Model 1 and Model 2 in ***3.5*** section: “Model 1 included known risk factors for T2DM, such as age, BMI, lipid profiles, creatinine levels, potassium, and hypertension. Model 2 incorporated all variables from Model 1, along with RAAS components, to adjust for their internal interactions. Subsequently, efficacy subgroups were established to assess the effects of dapagliflozin.”

We have revised “independent of RAAS components” as “after additional adjustment for Log-REN and Log-AII”, for example: In ***3.5*** section: “In the DKD cohort, after additional adjustment for Log-REN and Log-AII (Model 2), a 100% increase in Log-ALD was associated with a 0.143 nmol/L higher CP (95% CI: 0.002~0.283, p=0.047) and a 15.8% higher HOMA-IR (95% CI: 2.5%~29.0%, p=0.020). After 3 months of treatment, these correlations were markedly diminished and no longer statistically significant (p=0.582 and 0.634, respectively; Model 2).”

**C20:** Specify whether adjustments for multiple comparisons (e.g., Bonferroni correction) were applied to control for type I errors. Include effect sizes for significant findings to provide context for their magnitude. Briefly explain the implications of log-transformation for regression analyses (e.g., how it affects the interpretation of β coefficients).

**A20:** Great thanks for your comment. This study primarily involved comparisons between two independent samples and paired data. Multiple linear regression analysis was performed using the "Enter" method in two covariate models, with adjustments made for multiple variables, so multiple comparisons were unnecessary.

Discussion and conclusion:
**C21:** The discussion mentions that SGLT-2is "weakened the correlations between RAAS and glucose metabolism disorders," but the specific mechanisms underlying this observation are not clearly elucidated. The authors should provide a more detailed explanation of how these correlations are mitigated and whether this is a direct or indirect effect of SGLT-2is.

**A21:** Great thanks for your comment. The discussion has been appropriately revised in the ***fifth*** paragraph of the discussion, as follows: “Our prospective study firstly found that SGLT-2 inhibitors reduced the correlation between RAAS and glucose metabolism. After three months, RAAS markers (AII, REN, and ALD) returned to baseline, and glucose-related indicators decreased significantly, suggesting SGLT-2 inhibitors indirectly mitigate RAAS-glucose metabolism interplay by lowering blood glucose levels [1].”

**C22:** The discussion presents conflicting evidence about SGLT-2is’ effects on RAAS (e.g., no significant effect on aldosterone [ALD] or REN in some studies [17], versus increased REN and reduced ARR in others [14, 16]). While this reflects the controversy, the authors do not sufficiently reconcile these discrepancies or propose hypotheses for why these differences exist (e.g., differences in study design, patient populations, or SGLT-2i agents).

**A22:** Great thanks for your comment. The discussion has been appropriately revised in the ***second*** paragraph of the discussion, as follows: “This heterogeneity arose from variations in study designs (e.g., inclusion criteria, follow-up periods), control conditions (such as self-control), diets (including sodium and protein intake), sampling strategies (e.g., consecutive, random, or convenience sampling), medication dosages, and treatment adherence [10, 13, 25].”

**C23:** The study focuses on short-term (1-month) effects of dapagliflozin, but the discussion only briefly mentions that REN and ALD levels stabilize with long-term use (>3 months). A more robust discussion of how short-term versus long-term effects might influence clinical decision-making (e.g., timing of PA screening) would strengthen the manuscript.

**A23:** Great thanks for your comment. The discussion has been appropriately revised in the ***second*** and ***third*** paragraphs of the discussion, for example: “Interestingly, no temporal differences were observed in ALD levels. REN levels returned to baseline after three months of treatment; yet the ARR, which was primarily influenced by REN, remained below its initial value. This result indicated that the ARR, calculated using ALD and REN levels, was more sensitive to the effects of drug treatment. A slight increase in renin resulted in a significant decrease in the ARR. Consequently, clinicians should exercise caution when interpreting ARR in patients with type 2 diabetes mellitus (T2DM) undergoing short-term SGLT-2 inhibitor therapy due to the risk of false-negative primary aldosteronism (PA) screening.”

“Multiple studies have investigated the timeline for fluid volume recovery following the discontinuation of SGLT-2is. The EMPA-REG outcome study indicated that the estimated glomerular filtration rate (eGFR), an indicator of fluid volume recovery, returned to baseline at a median of 34 days after stopping SGLT-2is [29]. Therefore, discontinuing SGLT-2is for 4-5 weeks may be advisable before measuring the ARR. However, the optimal timing for medication withdrawal prior to ARR requires further validation.”

**C24:** While limitations are acknowledged, the discussion does not adequately address their implications. For example, the lack of PA patients is a significant limitation, as the study’s findings on ARR and false-negative PA screening are speculative without direct evidence from PA patients. The authors should discuss how this limits the generalizability of their findings.

**A24:** Great thanks for your comment. The discussion has included the limitation in the ***sixth*** paragraph, for example: “Secondly, our study did not include patients with PA; instead, we selected participants at risk for PA, specifically those with T2DM and hypertension. This approach provided the opportunity to assess whether SGLT-2is increase the risk of false-negative screening results [10], as most existing researches on drug interference with ARR have been conducted on patients without PA [10, 36, 37].” “Consequently, further researches to support our findings are warranted and should include patients with confirmed PA”

**C25:** The claim that SGLT-2is and RAAS antagonists produce synergistic effects [28, 39-41] is not sufficiently supported by the study’s data, as the study did not directly investigate combination therapy. This statement should be tempered or supported with more specific evidence from the current study.

**A25:** Great thanks for your comment. We have removed this part of the discussion.

**C26:** The conclusion is concise but could be more impactful by summarizing the clinical implications more explicitly. For example, state that “clinicians should be cautious when interpreting ARR in T2DM patients on short-term SGLT-2i therapy due to the risk of false-negative PA screening.

**A26:** Great thanks for your comment. We have revised the conclusions as follows: “SGLT-2is treatment may indirectly weaken the correlation between RAAS and glucose metabolism disorders by reducing blood glucose levels. However, short-term therapy (≤3 months) may elevate REN levels and reduce ARR. Consequently, clinicians should exercise caution when interpreting ARR in T2DM patients on short-term SGLT-2is therapy due to the risk of false-negative PA screening.”

**C27:** Highlight the need for future research to determine the optimal duration for discontinuing SGLT-2is before PA screening and to validate these findings in PA patients.

**A27:** Great thanks for your comment. We have revised the conclusions as follows: “Further studies to determine the optimal duration for discontinuing SGLT-2is before ARR test are warranted and should validate these findings in PA patients.”
