# Supplementary material for: Effects of SGLT-2 inhibitors on renin-angiotensin-aldosterone system and their correlation with glucose metabolism in type 2 diabetes mellitus patients with hypertension: A prospective study
Source: PLoS One. 2025 Nov 18;20(11):e0336158. doi: 10.1371/journal.pone.0336158 (PMC12626264; doi:10.1371/journal.pone.0336158)
Supplement: Attachment — Submitted filename: Response to reviewers.docx [file pone.0336158.s006.docx]

**2025.09.26**

Dear Dr. Zhanjun Jia:

**Re:** **PONE-D-25-15425R1**

**Title:** Effects of SGLT-2 inhibitors on renin-angiotensin-aldosterone system and their correlation with glucose metabolism in type 2 diabetes mellitus patients with hypertension: A prospective study

Thank you for your kind letter of “**PLOS ONE PONE-D-25-15425R1 Revision Request**” on **September 18, 2025**. We thank the editorial requests for the corrections, and we deeply apologized for the considerable inconvenience caused to you due to our errors exist. We have now revised the manuscript according to the concerns raised in the reviews. A detailed copy of the authors' “point-by-point response to the reviewer's comments” is attached, and specific changes addressing reviewers' comments are marked.

Again, we would like to thank you for taking the time to review our manuscript. We would be most grateful for your kind consideration of this revised manuscript for potential publication in ***PLOS ONE***.

We look forward to hearing from you at your earliest convenience.

Sincerely yours,

Ningning Wang

Corresponding author:

Baohong Yue, Professor, PhD

Address: Department of Laboratory Medicine, The First Affiliated Hospital of Zhengzhou University, Zhengzhou, P. R. China

**Responses to the Reviewer’s comments:**

**Re: PONE-D-25-15425R1**

**Title:** Effects of SGLT-2 inhibitors on renin-angiotensin-aldosterone system and their correlation with glucose metabolism in type 2 diabetes mellitus patients with hypertension: A prospective study

We would like to acknowledge the reviewer’s insightful comments and valuable suggestions. In addition, we thank the reviewer for helping us improve the present manuscript. We have addressed the concerns raised by the reviewer as detailed below.

**Reviewer #2**

**C1:** The extension of follow-up from one month to three months substantially strengthens the study’s validity and addresses earlier concerns regarding short-term variability in RAAS parameters.

**A1:** We appreciate the favorable comments provided by the reviewer.

**C2:** Clarifying the sampling method and recruitment period enhances reproducibility and transparency.

**A2:** We appreciate the favorable comments provided by the reviewer.

**C3:** The explicit description of inclusion/exclusion criteria and medication washout timelines reduces ambiguity in patient selection.

**A3:** We appreciate the favorable comments provided by the reviewer.

**C4:** Inclusion of sample size estimation addresses concerns about statistical power.

**A4:** We appreciate the favorable comments provided by the reviewer.

**C5:** Discuss more explicitly the clinical implications of persistent UACR elevation in DKD patients post-treatment.

**A5:** We appreciate the favorable comments provided by the reviewer.

**C6:** Consider adding a limitations paragraph addressing residual confounding due to convenience sampling and single-center design.

**A6:** Great thanks for your comment. We have added a limitation in the ***Discussion*** as follows “Finally, this study recruited patients through convenience consecutive sampling at a single center. Despite employing robust statistical techniques, the possibility of residual, unmeasured confounding factors cannot be excluded. Consequently, further prospective multicenter studies with larger sample size are warranted and should include patients with confirmed PA.”

**Reviewer #4**

A) Executive Summary and Overall Recommendation

**C1:** Study focus: Prospective, pre–post evaluation of SGLT-2 inhibitor (dapagliflozin) effects on RAAS components (aldosterone, renin, aldosterone–renin ratio [ARR]) and their relationships with glucose metabolism among adults with type 2 diabetes and hypertension, including DKD vs. non-DKD subgroups.

Main findings: Within 1–3 months of treatment, renin increased, ARR decreased, and glycemic indices improved. Authors warn about potential false-negative primary aldosteronism (PA) screening shortly after initiating SGLT-2 inhibitors.

Verdict: Promising and clinically relevant dataset, but requires major revisions to resolve methodological inconsistencies (treatment duration; medication washout), clarify ARR computation/units, specify statistical models and multiplicity control, ensure consistent terminology, and clean language/formatting.

**A1:** We appreciate the favorable comments provided by the reviewer. We have addressed the concerns raised, as detailed below.

B) Major Scientific and Statistical Issues (Actionable)

**C2:** Internal inconsistency in intervention period

Abstract and Results imply a 3‑month protocol, while Methods elsewhere state “one month” and then analyze baseline vs 1 and 3 months. Reconcile everywhere (Abstract, Methods–Intervention, Figure captions, Limitations/Conclusions). State the exact dosing schedule and assessment timepoints (baseline, 1 month, 3 months).

**A2:** Great thanks for your comment. We have removed tracked-change residues and standardized the dosing schedule (3 months) and assessment timepoints (baseline, 1 month, 3 months) consistently throughout the entire text.

**C3:** ARR units and computation ARR

ARR is unitless. Remove units (e.g., “ARR −0.59±1.19 ng/L” is incorrect). Specify assays and units for aldosterone and renin (e.g., direct renin concentration vs plasma renin activity), justify any scaling (ARR = ALD/(REN×10)), and provide references. Use the same system when interpreting PA screening. ARR

**A3:** Great thanks for your comment. We apologize for our errors exist, and have removed the incorrect ARR units in the ***Abstract*** and Section ***3.3***. As stated in reference 8, when ALD was measured in ng/dL and REN in ng/L, the common cut-off value for the ARR was around 5.7(refer to the table below). In our study, both REN and ALD were measured in ng/L. Since 1 ng/L equals 10 ng/dL, the ARR was calculated using the formula: ARR = ALD / (REN × 10).

**C4:** Primary outcome, multiplicity, and model specification

Declare a single primary endpoint (e.g., ΔARR at 1 month) and the primary contrast (within‑patient change; or difference‑in‑change DKD vs non‑DKD). Address multiplicity (Holm/FDR) across multiple endpoints/timepoints. Provide full model formulas with covariates and diagnostics (normality of residuals, linearity, collinearity).

**A4:** Great thanks for your comment. We have added the primary and secondary outcomes in ***Abstract*** and Section ***2.3***: “The primary outcome measure was the change in renin (REN) levels during outpatient visits at baseline, 1 month, and 3 months. The secondary outcome was the change in other metabolic biomarkers at baseline and 3-month visit.”

The multiplicity across multiple timepoints was adjusted using the Bonferroni test, with the significance level adjusted to 0.017. Therefore, we have revised the significance level indicated by the asterisk in the legends of Figure 3 and Table 3 from “**p* < 0.05 vs. baseline” to “**p* < 0.017 vs. baseline”. The ***Abstract*** and Section ***3.3*** of the ***Results*** have also been appropriately revised. The full models with covariates (Model 1 and Model 2) have already included in Sections ***2.4*** and ***3.5***.

**C5:** Causal wording vs observational design

Reframe as association given the single‑arm pre/post design. Avoid mechanistic claims (e.g., “SGLT‑2i weakens RAAS–glucose coupling”); prefer “correlations attenuated after treatment.”

**A5:** Great thanks for your comment. We have revised the statement as “The correlations between RAAS markers and glucose metabolism indices were significantly attenuated, potentially due to lowered blood glucose levels after SGLT‑2is treatment” in the ***Abstract***, ***Discussion*** and ***Conclusions***.

**C6:** Sample size justification linkage

Power calculation targets a between‑group renin difference; the main analyses include within‑subject changes and regression models. Align the power target with the declared primary outcome/contrast and cite the source for Δ and SD assumptions.

**A6:** Great thanks for your comment. The reference [24] for “the source for mean difference and SD assumptions” has been added to Section ***2.4*** of the ***Methods***.

**C7:** Handling of non‑normality and dispersion

Outcomes such as UACR display heavy tails (very large SDs). Provide median (IQR) alongside mean (SD); consider robust regression or transformation. State normality checks; default to non‑parametric tests where appropriate.

**A7:** Great thanks for your comment. Continuous variables that followed non-normal distribution (Diabetic duration, BMI, TG, AII, ALD, REN, ARR, UACR, CP, HOMA-β, HOMA-IR) were presented as median with interquartile range in Tables 1, 3, S1, S2 and S3. These non-normally distributed data were analyzed using non-parametric tests.

Tables S1 and S2 presented the specific biochemical indicator values (including UACR, presented as median with IQR) before and after treatment for both groups. The results of the statistical analysis were consistent with those shown in Table 2.

Moreover, the non-normally distributed variables (AII, ALD, REN, ARR, UACR, CP, HOMA-β, HOMA-IR) were log-transformed to perform multiple linear regression analyses.

**C8:** Terminology consistency

Standardize terms: DKD (not intermixing DN), ARR (not AAR), fasting blood glucose as FBG (avoid GLU in places). Ensure figure/legend labels match text.

**A8:** Great thanks for your comment. We have removed all inconsistent terminology and tracked‑change residues from the entire manuscript. The modified parts have been highlighted.

Discussion/Conclusions

**C9:** Tone down causal attributions; emphasize concurrent changes and potential confounding. Clarify the clinical caution about PA screening in the context of your precise ARR computation/assay and standard cut‑offs. Expand limitations (single center, washout realities, single‑arm design, regression to the mean).

**A9:** Great thanks for your comment. We have toned down causal attributions; for example, we changed “SGLT‑2i weakens RAAS–glucose coupling” to “correlations were attenuated after treatment.”

We have added a limitation in the ***Discussion*** as follows “Finally, this study recruited patients through convenience consecutive sampling at a single center. Despite employing robust statistical techniques, the possibility of residual, unmeasured confounding factors cannot be excluded. Consequently, further prospective multicenter studies with larger sample size are warranted and should include patients with confirmed PA.”

**C10:** Ethical clarity on concomitant therapies

RAAS endpoints are sensitive to ACEi/ARB/mineralocorticoid antagonists. Clarify whether these were stopped and for how long; provide baseline concomitant medications by subgroup and discuss potential residual effects.

**A10:** Great thanks for your comment. The inclusion criteria in Section ***2.2*** of the ***Methods*** already specified the concomitant drug conditions as follows“Patients had not taken any agents that could markedly affect the ARR, such as angiotensin converting enzyme inhibitors, angiotensin receptor blockers, diuretics, or calcium channel blockers within the last 2 months, or aldosterone receptor antagonists, β-blockers, or direct renin inhibitors within the last 4 months [8]; For patients with poorly controlled blood pressure (≥160/100 mmHg), medications with less effect on RAAS , such as α-blockers (e.g., terazosin), were administered.”

C) Figures and Tables – Technical Assessment

**C11:** Tables: In the outcomes table (e.g., Δ from baseline), clarify that asterisks denote within‑group change vs baseline, whereas the p‑value column compares Δ between groups. Specify tests used per row (paired t/Wilcoxon; two‑sample t/Mann–Whitney) and show units (e.g., UACR mg/g). Ensure consistent decimal precision and spacing around ±.

**A11:** Great thanks for your comment. Explanations for the above questions have been added to the labels in Table 2 as follows “Tables S1 and S2 presented the specific values and the results of the statistical analysis before and after treatment for both groups. Asterisk denotes within‑group change vs. baseline (^a^, Wilcoxon matched pairs signed rank test; the other rows: paired *t*-test); the p‑value column compares the difference between DKD and non‑DKD (two‑sample *t-*test). (**p*<0.05 baseline vs. 3 months)” The unit of UACR has also been added.

**C12:** Figures: Ensure axis units are shown for RAAS markers; define error bars (SD vs SEM vs 95% CI); label DKD vs non‑DKD and timepoints consistently. Clean legends (remove duplicated text, typos) and align terminology across text and graphics. Provide cost‑effectiveness style plots only if relevant; otherwise, keep to biological/clinical outcomes.

**A12:** Great thanks for your comment. We have defined the error bars in the legend of Fig. 3 as follows: “Data are expressed as median with interquartile range.” We have removed all inconsistent terminology and tracked‑change residues from the legends.

D) Line-Referenced Language and Formatting Corrections

Below are concrete edits. Where exact line numbers vary across versions, use the section/phrase to locate the sentence.

**C13:** Abstract – Results sentence with ARR units

“ARR decreased by −0.59±1.19 ng/L …” → “ARR decreased (mean Δ −0.59±1.19; unitless).” Remove units; ARR is a ratio.

**A13:** Great thanks for your comment. We apologize for our errors exist, and have removed the incorrect ARR units.

**C14:** Abstract – Wording

Replace “ditto; p>0.05” with “between‑group difference was not statistically significant (p>0.05).”

**A14:** Great thanks for your comment. We have replaced “ditto; *p*>0.05” with “between‑group difference, *p*>0.05.” in the ***Abstract***.

**C15:** Methods – Intervention duration

Replace conflicting statements (“one month” vs “three months”) with the final, consistent schedule: “Participants received dapagliflozin for 3 months; assessments occurred at baseline, 1 month, and 3 months.”

**A15:** Great thanks for your comment. We have removed tracked-change residues and standardized the dosing schedule (3 months) and assessment timepoints (baseline, 1 month, 3 months) consistently throughout the entire text.

**C16:** Methods – ARR formula/units

After “ARR = ALD/(REN×10),” specify ALD and REN assays and units, justify the ×10 scaling with a reference, and remove units when reporting ARR values.

**A16:** Great thanks for your comment. We apologize for our errors exist, and have removed the incorrect ARR units. As stated in reference 8, when ALD was measured in ng/dL and REN in ng/L, the common cut-off value for the ARR was around 5.7(refer to the table below). In our study, both REN and ALD were measured in ng/L. Since 1 ng/L equals 10 ng/dL, the ARR was calculated using the formula: ARR = ALD / (REN ×10).

**C17:** Eligibility – Washout windows and examples

Ensure drug classes and washout intervals are consistent (e.g., ACEi/ARB/diuretics/CCB: ≥2 months; MRA/β‑blockers/direct renin inhibitors: ≥4 weeks), and correct “trazodone” to “terazosin” as the α‑blocker example.

**A17:** Great thanks for your comment. We have ensured that drug classes and washout intervals are consistent and corrected “trazodone” to “terazosin” as the α‑blocker example in Section ***2.2*** of ***Methods***.

**C18:** Results – Table notes

In the outcomes table, add: “Asterisk denotes within‑group change vs baseline; p‑value compares change between DKD and non‑DKD.” Add units for UACR (mg/g) and maintain consistent decimals.

**A18:** Great thanks for your comment. Explanations for the above questions have been added to the labels in Table 2 as follows “Tables S1 and S2 presented the specific values and the results of the statistical analysis before and after treatment for both groups. Asterisk denotes within‑group change vs. baseline (a, Wilcoxon matched pairs signed rank test; the other rows: paired t-test); the *p*‑value column compares the difference between DKD and non‑DKD (two‑sample t-test). (**p*<0.05 baseline vs. 3 months)” The unit of UACR has also been added.

**C19:** Discussion – Causal phrasing

Change “SGLT‑2 inhibitors weaken the RAAS–glucose interaction” to “After SGLT‑2 inhibitor initiation, correlations between RAAS markers and glycemic indices were attenuated.”

**A19:** Great thanks for your comment. We have revised the statement as “The correlations between RAAS markers and glucose metabolism indices were significantly attenuated, potentially due to lowered blood glucose levels after SGLT‑2is treatment” in the ***Abstract***, ***Discussion*** and ***Conclusions***.

**C20:** Limitations – Typos/artifacts

Correct artifacts like “3one‑month,” “threewo time points,” and fused words (e.g., “slowingdelaying”). Standardize to “slowing or delaying.”

**A20:** Great thanks for your comment. We have removed all inconsistent terminology and tracked‑change residues from the entire manuscript. The modified parts have been highlighted.

**C21:** Global – Terminology

Use DKD (not DN), ARR (not AAR), FBG (avoid GLU). Ensure consistency in figures/legends/tables and across text.

**A21:** Great thanks for your comment. We have removed all inconsistent terminology and tracked‑change residues from the entire manuscript. The modified parts have been highlighted.

**C22:** Global – Formatting

Remove tracked‑changes residues and “Formatted:” comments; standardize spacing around ± and between numbers/units; ensure subject‑verb agreement in Statistics.

**A22:** Great thanks for your comment. We have removed all inconsistent terminology and tracked‑change residues from the entire manuscript. The modified parts have been highlighted.
